# PET Diagnostic Molecules Utilizing Multimeric Cyclic RGD Peptide Analogs for Imaging Integrin α_v_β_3_ Receptors

**DOI:** 10.3390/molecules26061792

**Published:** 2021-03-22

**Authors:** Christos Liolios, Christos Sachpekidis, Antonios Kolocouris, Antonia Dimitrakopoulou-Strauss, Penelope Bouziotis

**Affiliations:** 1 Radiochemical Studies Laboratory, Institute of Nuclear & Radiological Sciences & Technology, Energy & Safety, National Centre for Scientific Research “Demokritos”, Ag. Paraskevi Attikis, 15310 Athens, Greece; 2 Laboratory of Medicinal Chemistry, Department of Pharmacy, Section of Pharmaceutical Chemistry, National and Kapodistrian University of Athens, Panepistimioupolis–Zografou, 15771 Athens, Greece; ankol@pharm.uoa.gr; 3 Clinical Cooperation Unit Nuclear Medicine, German Cancer Research Center (DKFZ), 69120 Heidelberg, Germany; c.sachpekidis@dkfz-heidelberg.de (C.S.); a.dimitrakopoulou-strauss@dkfz-heidelberg.de (A.D.-S.)

**Keywords:** PET imaging, multimeric radioligands, integrin α_v_β_3_, cyclic RGD

## Abstract

Multimeric ligands consisting of multiple pharmacophores connected to a single backbone have been widely investigated for diagnostic and therapeutic applications. In this review, we summarize recent developments regarding multimeric radioligands targeting integrin α_v_β_3_ receptors on cancer cells for molecular imaging and diagnostic applications using positron emission tomography (PET). Integrin α_v_β_3_ receptors are glycoproteins expressed on the cell surface, which have a significant role in tumor angiogenesis. They act as receptors for several extracellular matrix proteins exposing the tripeptide sequence arginine-glycine-aspartic (RGD). Cyclic RDG peptidic ligands c(RGD) have been developed for integrin α_v_β_3_ tumor-targeting positron emission tomography (PET) diagnosis. Several c(RGD) pharmacophores, connected with the linker and conjugated to a chelator or precursor for radiolabeling with different PET radionuclides (^18^F, ^64^Cu, and ^68^Ga), have resulted in multimeric ligands superior to c(RGD) monomers. The binding avidity, pharmacodynamic, and PET imaging properties of these multimeric c(RGD) radioligands, in relation to their structural characteristics are analyzed and discussed. Furthermore, specific examples from preclinical studies and clinical investigations are included.

## 1. Introduction

### 1.1. Multimeric Radioligands

**Figure 1:**
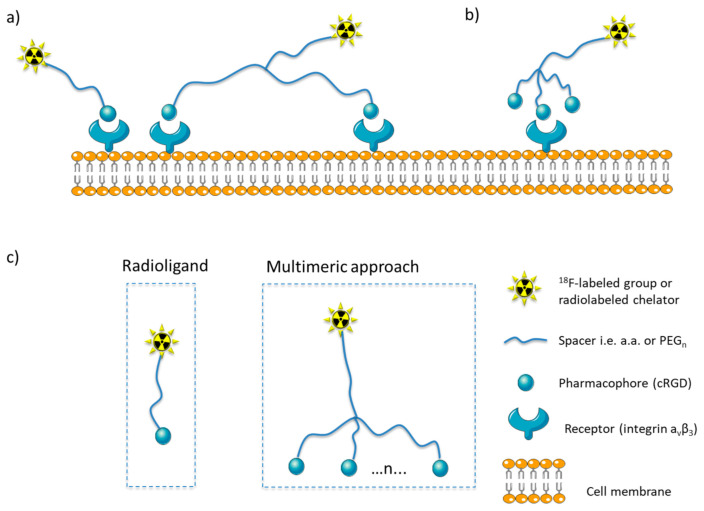
Binding models for multimers on the cell surface: (**a**) The binding of a radioligand to a cell surface receptors and the multimeric approach resulting in simultaneous binding of two pharmacophores connected via a long linker with two receptors, (**b**) improved binding efficiency of a ligand, due to the increased apparent local concentration of the pharmacophore (statistical effect) in the micro-environment of the receptor; (**c**) basic principles for the design of monomeric and multimeric radioligands (where *n* = number of pharmacophores).

The idea of multimeric radioligand, which consists of multiple structural motifs connected to a single backbone, started from the need to achieve higher binding avidity through cooperative multivalent interactions. In a way, this mimics the behavior of antibodies, which have multiple individual weak interactions with the target. These multiple weak interactions combine in a cooperative way and increase binding avidity [1,2]. Several binding modes have been proposed as an explanation for this cumulative effect, such as the simultaneous binding of the ligand to multiple receptors on the cell surface (Figure 1a), or the improved statistical effect, where the ligand binds to one receptor, but the ligand’s apparent local concentration is increased (Figure 1b) [1].

Multimeric radioligands resemble a typical radioligand by retaining some of its structural features—e.g., a chelator group for radiometals—and differ from it, as they include multiple pharmacophores. Radioligands targeting specific receptors on tumor cell surfaces consist of a receptor binding section (pharmacophore or ligand), and a radiolabeled domain. The latter is either a radiometal chelator, which coordinates with a radiometal, or a suitable group for radio-halogenation, which is often connected with the pharmacophore through a linker (spacer) group. Linker groups are used (Figure 1): (1) to increase the distance between the ligand and the chelator group and thus, minimize the interference of the radiolabeled domain with the binding pocket of the ligand in the receptor; (2) to enhance the interactions between the ligand and its binding pocket; (3) to enable the addition of a second or of multiple binding pharmacophore groups leading to multimeric ligands; (4) to increase the distance between the multiple pharmacophores in order to enable simultaneous binding on more than one receptors (Figure 1a); (5) to change the pharmacokinetic properties of the radioligand, altering its hydrophobicity and eventually increase its apparent local concentration when it reaches the receptor’s binding area [3,4,5].

The linker properties are very important since they influence the orientation of the ligand, when it approaches the extracellular receptors binding domain, enabling or preventing simultaneous multi-receptor binding of the multimers [6]. Long, flexible linkers increase the distance between the binding domains, permitting simultaneous binding of the ligand to multiple receptors, while rigid linkers have less entropy penalty from binding [1].

For multimeric radioligands, a wide spectrum of pharmacophores has been used like small molecules, peptides, or fractions of antibodies [1,6,7,8,9]. A limited number (*n* = 2–8) of pharmacophores e.g., c(RGD)_n_ has been covalently connected using low molecular weight linkers e.g., polyethylene glycols (PEGs), while higher numbers of pharmacophores have been tethered on the surface of large molecules, such as liposomes or nanoparticles. The nanoparticle approach is not examined in this review.

### 1.2. Multimerization of Cyclic RGD Peptides

Angiogenesis is a fundamental process required for tumor survival, proliferation, and progression [10]. Among the factors regulating angiogenesis are the proteins known as integrins [11,12]. Overexpression of integrins on cancer cells potentiates metastasis by facilitating their invasion and movement across the blood vessels [13]. In particular, integrins α_v_β_3_ are considered an ideal target for the development of radioligands, since they are overexpressed on tumor vasculature due to angiogenesis, and on the cell membrane in various tumors, i.e., ovarian cancer [14], neuroblastoma [15], breast cancer [16,17,18] and melanoma [19]. Integrins α_v_β_3_ are transmembrane receptors for several extracellular matrix proteins exposing the tripeptide sequence arginine-glycine-aspartic (RGD). The tripeptide RGD amino acid sequence specifically binds to the integrin α_v_β_3_ receptor and this has also provided the platform for the development of cyclic RGD, c(RGD), radioligands [20].

The incorporation of the RGD sequence into a cyclic pentapeptide: cyclo-(Arg-Gly-Asp-d-Phe-Val), c(RGDfV), has been found to increase binding affinity and selectivity for integrin α_v_β_3_ receptor over glycoprotein IIb/IIIa (also known as integrin α_IIb_β_3_) [20]. After extensive structure-activity evaluations, it was concluded that the 5th amino acid has no significant impact on integrin α_v_β_3_ binding affinity. Thus, the valine residue in c(RGDfV) was replaced by lysine or glutamic acid to afford cyclo-(Arg-Gly-Asp-d-Phe-Lys) or cyclo-(Arg-Gly-Asp-d-Tyr-Glu) c(RGDfK) and c(RGDfE), respectively, without changing the integrin α_v_β_3_ binding affinity. The replacement of the 5th amino acid was crucial since it enabled further modification (e.g., conjugation to a chelator group) via the formation of peptide bonds with the side group of K or E, respectively [20].

Multimeric c(RGD) radioligands consist of several binding domains: c(RGDfK), c(RGDfE), c(RGDyK), tethered together via some sort of backbone e.g., c(RGDfK)-X-c(RGDfK) (X = linker). In low molecular weight multimers, X is composed of amino acids: Glu (E), Lys (K), enabling the installation of the radiolabeled section with a chelator group and multiple binding domains. Sometimes a polyethylene glycol unit PEG_n_ (*n* = number of polyethylene glycol units) is also introduced to increase the distance between the pharmacophores and the radiolabeled domain [20,21]. For example, if X = Glu (E) multimerization can be accomplished by connecting its alpha -NH_2_ with a chelator while conjugating with peptide bonds the alpha -COOH and the side chain -CH_2_COOH, with the 5th amino acid (K) of two c(RGDfK) pharmacophores. The linkage structure plays a critical role in the pharmacodynamic properties of the c(RGDfK)_n_ multimeric ligands e.g., attaching a long PEG chain to the amino acid linker has proved advantageous for enhancing avidity and tumor uptake. The synthesis of such molecules is usually accomplished using orthogonal, sequential solid-phase peptide synthesis (spps), liquid phase peptide synthesis [20,22], or conjugation of the different parts using copper-catalyzed azide-alkyne cycloaddition (CuAAC) [23].

Based on this framework, a variety of radiolabeled cRGD peptides has been developed for non-invasive imaging of integrin α_v_β_3_ expression with PET, e.g., [^18^F]FP-PRGD_2_ or [^18^F]FP-PEG_3_-E[c(RGDyK)]_2_ **8**, [^18^F]Alfatide I or [^18^F]AlF-NOTA-PEG_3_-E[c(RGDyK)]_2_ **10**, [^18^F]Alfatide II or [^18^F]AlF-NOTA-E[PEG_4_-c(RGDfK)]_2_ **14**; [^68^Ga]Ga-NOTA-PEG_4_-E[c(RGDfK)]_2_ **12**, [^68^Ga]Ga-NOTA-E[PEG_4_-c(RGDfK)]_2_, or [^68^Ga]Ga-NOTA-PRGD_2_ **15** and [^64^Cu]Cu-NOTA]-PEG_4_-cRGD_2_ or [^64^Cu]Cu-DOTA-E(E{E[c(RGDyK)]_2_}_2_)_2_ **28** (where NOTA = 1,4,7-triazacyclononane-1,4,7-triacetic acid, PEG_3_ = 12-amino-4,7,10-trioxadodecanoic acid, PEG_4_ = 15-amino-4,7,10,13-tetraoxapentadecanoic acid) [24,25,26]. The various pharmacophores: c(RGDfK), c(RGDyK), c(RGDfE), are all confusingly abbreviated in the literature as RGD_v_ (v = number of pharmacophores), and with the addition of a PEG linker as PRGD_v_ independently of the PEG length. Among the multimers hitherto evaluated in preclinical studies, the dimeric structures are the most successful. The majority of these dimers has already advanced in clinical oncological studies (**8**, **10**, **12**, **14**, **15**) while some of them have also been studied for other pathological conditions apart from cancer e.g., [^18^F]FP-PRGD_2_ or [^18^F]FP-PEG_3_-E[c(RGDfK)]_2_ **8** for the prediction of fibrosis in non-alcoholic steatohepatitis [27]. Several groups prepared c(RGD) tetramers [28,29] and octamers [30] based on the observation that pharmacophore multiplicity may increase the integrin α_v_β_3_ binding avidity and ligand cell-internalization [30]. However, none of these multimers has advanced to clinical studies. The goal of this review is to summarize recent developments of c(RGD)_n_ multimeric radioligands, which target the integrin α_v_β_3_ receptors, for cancer diagnosis using positron emission tomography (PET) molecular imaging.

## 2. Preclinical Studies of Multimeric Cyclic RGD Peptides

### 2.1. ^18^F Labeled Cyclic RGD Multimers

The majority of PET imaging preclinical and clinical studies refer to ^18^F labeled c(RGD)_n_ multimers, while following them are the ^68^Ga and ^64^Cu labeled ones. ^18^F is a PET radioisotope combining favorable physical and chemical characteristics (half-life = 109 min, β^+^ 96.7%, 0.64 MeV), which can be easily produced as aqueous fluoride by means of proton irradiation of ^18^O-enriched water with a biomedical cyclotron [31]. ^18^F-radiolabeling is achieved using reactions of nucleophilic substitution on suitable precursors, which carry appropriate leaving groups e.g., halides, sulfonates, and ammonium cations, or aromatic rings with activating residues like electron-withdrawing groups in appropriate positions [32].

**Figure 2:**
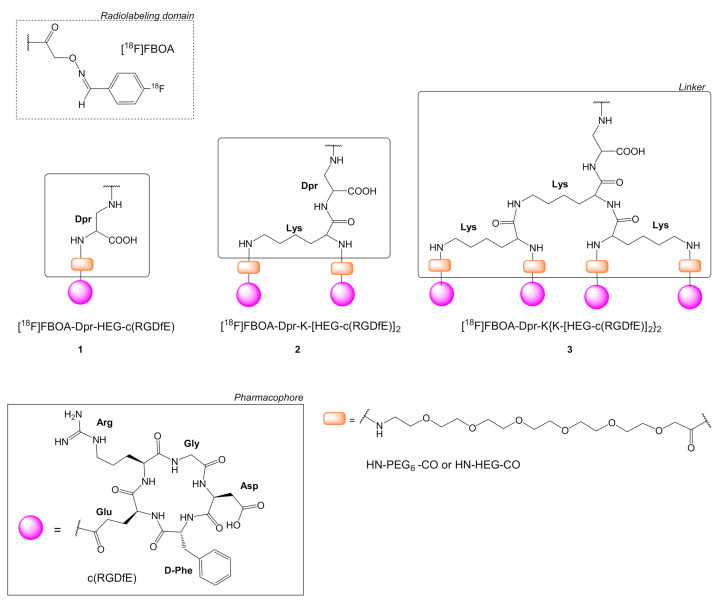
Chemical structures of c(RGDfE) peptides with a PEG_6_ linker (H_2_N-PEG_6_-CH_2_COOH); monomer [^18^F]FBOA-Dpr-HEG-c(RGDfE) **1**, dimer [^18^F]FBOA-Dpr-K(HEG-c(RGDfE))_2_ **2** and tetramer [^18^F]FBOA-Dpr-K{K[HEG-(c(RGDfE)]_2_}_2_ **3** are labeled with *N*-(4-[^18^F]fluorobenzylidene)oxime ([^18^F]FBOA) (Dpr = diaminopropionic acid).

Poethko et al. in one of the first studies on ^18^F-multimeric c(RGD) radioligands used 4-[^18^F]Fluorobenzaldehyde, which was conjugated to H_2_N-*O*-CH_2_-CO-NH-functionalized peptides forming an oxime *N*-(4-[^18^F]Fluorobenzylidene)oxime ([^18^F]FBOA). The pharmacophores were linked to [^18^F]FBOA through 2,3-diaminopropionic acid (Dpr) connected to a Lys linker. The alpha and side-chain -COOH groups of Lys were conjugated to the Glu of each pentapeptide c(RGDfE) pharmacophore using a PEG_6_ group (H_2_N-PEG_6_-CH_2_COOH). The [^18^F]FBOA-Dpr-HEG-c(RGDfE) monomer **1** was compare with the dimeric [^18^F]FBOA-Dpr-K(HEG-c(RGDfE))_2_ **2**, and tetrameric [^18^F]FBOA-Dpr-K{K[HEG-(c(RGDfE)]_2_}_2_ **3** structure (Figure 2) [22,33]. Biodistribution studies for c(RGDfE) derivatives **1**–**3** were performed in mice bearing human melanoma M21 xenographs, overexpressing α_v_β_3_ integrin and M21-L, weakly expressing α_v_β_3_ integrin (negative control). Tumor uptake was higher for the dimeric structure **2** (60 min post-injection [p.i.]: 2.48 ± 0.15 percent of injected dose per gram [% ID/g]; 120 min p.i.: 1.63 ± 0.13% ID/g) compared to the monomer **1** (60 min p.i.: 1.56 ± 0.15% ID/g; 120 min: 1.49 ± 0.10% ID/g). Tumor uptake (M21 tumor) was higher by a factor of 5–6 than the negative control (M21-L), and specific, as it was proved by blocking studies. In another complementary study between ligands **1**–**3**, the tetrameric ligand **3** showed similar tumor uptake with **2** (120 min p.i.: 1.65 ± 0.08% ID/g), while its tumor-to-normal organ contrast ratios in all cases were better than both, **1** and **2**. Regarding normal organ biodistribution, **2** showed rapid renal excretion and low activity levels in blood, liver, and intestines. Renal excretion rates were initially comparable for both **1** and **2**, with monomer **1** showing higher excretion rates with time, while **2** remained in the kidneys. The tetrameric ligand, **3**, showed intermediate clearance kinetics, predominant renal excretion, and lower unspecific tissue distribution, while its liver uptake was lower than that of **1** and **2**. However, **3** showed significant metabolization, especially in liver, kidney, and tumor, which according to the authors was due to the effect of lysine linkers, which are present between PEG and Dpr groups, cleaving the ligand’s bridging to monomeric and dimeric units.

**Figure 3:**
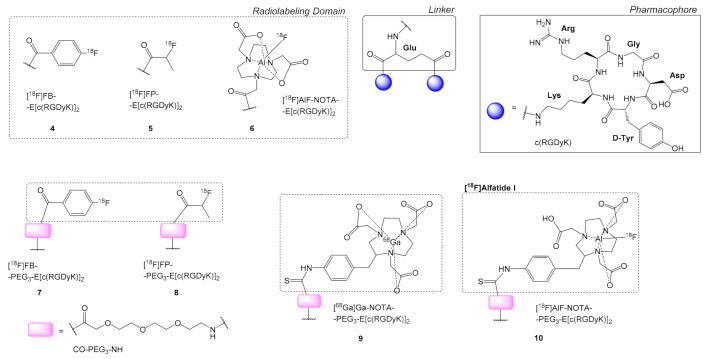
Chemical structures of dimeric radioligands containing the scaffold E(c(RGDyK)]_2_ without: [^18^F]FB-E[c(RGDyK)]_2_ **4**, and [^18^F]FP-E[c(RGDyK)]_2_ **5**, [^18^F]AlF-NOTA-E[c(RGDyK)]_2_ **6**, and with a PEG_3_ group in between the alpha H_2_N- group of E and the radiolabeled domain: [^18^F]FB-PEG_3_-E[c(RGDfK)]_2_ **7**, [^18^F]FP-PEG_3_-E[c(RGDfK)]_2_ **8**, [^68^Ga]Ga-NOTA-PEG_3_-E[c(RGDyK)]_2_ **9**, [^18^F]AlF-NOTA-PEG_3_-E[c(RGDyK)]_2_ or [^18^F]Alfatide I **10** (H_2_N-PEG_3_-COOH = 11-amino-3,6,9-trioxaundecanoic acid).

Chen et al. labeled with ^18^F the dimeric peptide E[c(RGDyK)]_2_ (Figure 3) acylating the alpha -NH_2_ group of the glutamate (E) linker (using *N*-succinimidyl 4-[^18^F]fluorobenzoate: [^18^F]SFB), **4**, while the alpha and side-chain -COOH groups were conjugated to the K of each pentapeptide c(RGDyK) [34]. The resulting dimeric peptide [^18^F]FB-E[c(RGDyK)]_2_, **4** showed higher uptake for primary human brain capillary endothelial cells (HBCECs) expressing integrin α_v_β_3_ (IC_50_ = 2.3 ± 0.7 nM) compared to the monomeric analogue [^18^F]FB-c(RGDyK) (IC_50_ = 3.5 ± 0.3 nM). When **4**, was administered to glioblastoma xenograft mouse model U87MG for micro-PET, autoradiographic imaging, direct tissue it showed significantly higher tumor uptake, and prolonged tumor retention in comparison to the monomer (120 min p.i. **4**: 4.27 ± 1.04% ID/g, monomer: 1.56 ± 0.35% ID/g). Additionally, the dimeric structure **4** showed predominant renal excretion, whereas the monomeric analog was excreted primarily through the biliary route, which during micro-PET imaging resulted in much higher tumor to contralateral background ratios. The superior imaging characteristics of **4** were attributed to the synergistic effect of poly-valency and improved pharmacokinetics [34].

The same dimeric scaffold E[c(RGDyK)]_2_ was also used for the synthesis of other multimers e.g., the [^18^F]FP-E[c(RGDyK)]_2_ **5**, in which the 2-^18^F-fluoropropionate ([^18^F]FP) is attached at the α-amino group of the glutamate linker moiety [35]. One of the major obstacles in the clinical translation of radioligands like **4** or **5**, is the laborious multistep ^18^F installation in the dimeric structure, which includes a four-step, two-pot procedure with a long reaction time [34,35]. In this context, Liu et al. [35] described the synthesis of [^18^F]AlF-NOTA-E[(c(RGDyK)]_2_ or [^18^F]AlF-NOTA-RGD_2_ **6**, (NOTA = 1,4,7-triazacyclononane-1,4,7-triacetic acid), using the Al^18^F intermediate (Figure 3) in a shorter time with good radiochemical yield (>17.9%). Integrin α_v_β_3_ binding avidity of **6** was assessed in the U87MG cell model resulting in an IC_50_ = 46 ± 4.4 nM. The tumor-targeting efficacy and in vivo profile of **6** and **5** were further evaluated in the same model by microPET and biodistribution. Interestingly, the two radioligands **5** and **6** had similar biodistribution and imaging properties, showing fast clearance from the body, and high tumor to normal organ contrast ratios. However, **6** showed slightly higher tumor uptake than **5** (120 min p.i.: 2.3 ± 0.9% ID/g instead of 1.3 ± 0.8% ID/g, respectively) [35].

### 2.2. PEG Linkers on ^18^F Labeled Cyclic RGD Multimers

Following the example of Poethko et al. [22,33] who introduced a long PEG_6_ group the Lys linker with the c(RGD) pharmacophores, shorter PEGs were introduced in between the linker and the labeling domain. The introduction of PEG spacer groups between the pharmacophores and the labeling domain improved the overall radiolabeling yield and the radioligands pharmacokinetics. Specifically, PEGs were introduced between the amino acid linker (E or K) and the radiolabeling domain and/or between the linker and the pharmacophores (Figure 3, **6**). For example, when a PEG_3_ was introduced in the structure of **4**, resulting in compound [^18^F]FB-PEG_3_-E(cRGDyK)_2_ or [^18^F]FB-PRGD_2_ **7** it increased the radiochemical yield (Yield > 60% and purity > 99%) [36]. In addition, **7** showed an improved pharmacological profile compared to **4**, since it showed higher receptor binding avidity **7** (IC_50_ = 40.6 ± 4.6 nM), **4** (IC_50_ = 55.1 ± 6.5 nM), higher metabolic stability, similar integrin α_v_β_3_-specific tumor uptake (U87MG glioma xenograft model, 30 min p.i.: 4.9–5% ID/g) and reduced renal uptake **7** (60 min: 2.0 ± 0.2% ID/g), **4** (60 min: 3.0 ± 0.2% ID/g). [36] The reproducibility of [^18^F]FP-PRGD_2_ **7** as an integrin α_v_β_3_-targeting PET probe was also verified using a small animal PET and mouse tumor xenograft (human HCT116 colon cancer) model. During those studies, **7** showed reproducible results with relatively low variability [37].

Rokugawa et al. investigated radioligand [^18^F]FP-PEG_3_-E[c(RGDyK)]_2_ **8** for PET scanning of fibrosis in non-alcoholic steatohepatitis (NASH) pathology through detecting hepatic integrin α_v_β_3_ expression in NASH [27]. C57BL/6 mice were fed with a choline-deficient, L-amino acid-defined, high-fat diet, which after some time (3, 8 weeks) produced moderate-to-severe steatosis and inflammation of the liver. PET scanning revealed that the hepatic uptake of **8** correlated well with integrin α_v_β_3_ expression and histological fibrosis in a mouse model of NASH, suggesting the predictability of fibrosis in NASH pathology [27].

Lang et al. compared [^18^F]FP-PEG_3_-E[c(RGDyK)]_2_ **8** with [^68^Ga]Ga-NOTA-PEG_3_-E[c(RGDyK)]_2_ **9** and [^18^F]AlF-NOTA-PEG_3_-E[c(RGDyK)]_2_ **10** (also called [^18^F]Alfatide) [38], where the NOTA chelator replaced the [^18^F]FP group enabling the labeling with a radiometal. Specifically, the *p*-SCN-Bn-NOTA chelator group (2-*S*-(4-Isothiocyanatobenzyl)-1,4,7-triazacyclononane-1,4,7-triacetic acid) was attached to the Glu linker via a PEG_3_ spacer resulting in a thioamide bond between PEG_3_ and NOTA (Figure 3). Radiolabeling procedure for **9** and **10** through complexation with ^68^Ga and Al^18^F, was much easier and resulted in high yields, i.e., **8** (10–15%), **9** (5–25%), **10** (75%) [38]. All three compounds **8**–**10** showed similar in vitro binding affinities (IC_50_ **8**: 175.4 nM, **9**: 119.2 nM, **10**: 82.7 nM), and in vivo rapid and high tracer uptake in U87MG tumors with high target-to-background ratios. Radioligands **9** and **10** showed imaging properties and pharmacokinetics comparable to **8** in U87MG-xenografted mice, with **10** showing slightly higher tumor uptake [38]. Complementary to the above results during quantitatively PET imaging studies (U87MG human glioblastoma) ligands **8**–**10** presented insignificant differences, although **10** again showed a slightly higher binding potential and specific distribution volume in the tumor [39].

[^18^F]Alfatide, **10** were studied as a predictor of anti-angiogenic response in alveolar adenocarcinoma (A549, high angiogenesis) and prostate cancer (PC-3, low angiogenesis) animal models, at baseline and after treatment with an anti-angiogenic drug (apatinib, bevacizumab) or without (control). The evaluation index for the inhibition of tumor growth in the individuals in the treated groups was represented by the treatment/control (T/C) ratio (%). Anti-angiogenic responses were denoted by the changes in uptake of **10** in the same animal. Uptake of **10** in the A549 models was significantly higher than in the PC-3 models (SUV_mean_ A549: 0.64 ± 0.07 and PC-3: 0.25 ± 0.02) and the same was true for T/N ratios (A549: 2.76 ± 0.62 and PC-3: 0.82 ± 0.11), before treatment. After anti-angiogenic treatment tumor growth was suppressed resulting in lower tumor/control values for the A549 xenografted tumors than the PC-3 tumors (apatinib, A549: 47 ± 11.46 and PC-3: 69 ± 26.74%; bevacizumab, A549: 57.80 ± 13.82 and PC-3: 90.27 ± 13.09%, respectively). In addition to the decreased [^18^F]Alfatide **10** uptake in tumors after treatment, the degree of tumor response was also associated with the tumor uptake prior to treatment, indicating that [^18^F]Alfatide PET may be a useful molecular imaging tool for individual patient selection prior to anti-angiogenic drug therapy [40].

In another study, **10** was stereotactically compared with ^18^F-fluorodeoxyglucose ([^18^F]FDG) and immunohistochemistry (IHC) staining in Lewis lung carcinoma (LLC) tumor-bearing C57BL/6 mouse model. Tumor-to-brain, tumor-to-lung, and tumor-to-heart ratios of **10** were significantly higher than those of [^18^F]FDG (*p* < 0.001). The spatial heterogeneity of the tumors was detected, and the tracer accumulation enhanced from the outer layer to the inner layer consistently. The different SUVs in the different tumor areas represent different levels of angiogenesis and probably deserve different radiation doses for tumor control. Correlations were found between: 1) SUV_RGD_ and the α_v_β_3_ expression in tumors (*R* = 0.595, *p* = 0.019) and 2) SUV_FDG_ and SUV_RGD_ (*R* = 0.917, *p* < 0.001). The latter corresponded to the IHC findings for the expression levels of glucose transporter proteins and α_v_β_3_, which were also correlated (*R* = 0.637, *p* = 0.011) [41].

**Figure 4:**
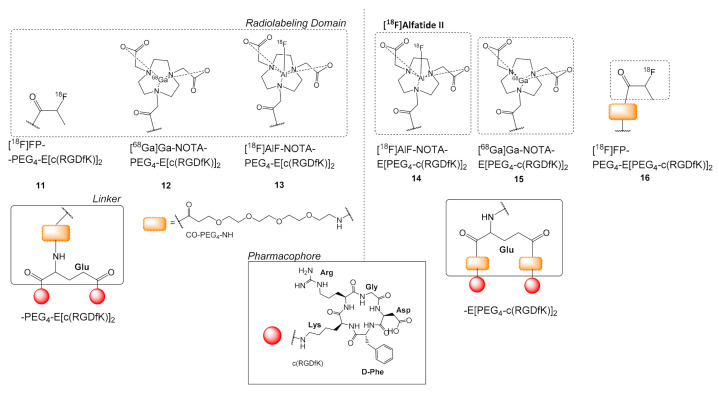
Chemical structures of c(RGDfK)]_2_ analogues with PEG_4_ spacers; [^18^F]FP-PEG_4_-E[c(RGDfK)]_2_ **11**, [^68^Ga]Ga-NOTA-PEG_4_-E[c(RGDfK)]_2_ **12**, [^18^F]AlF-NOTA-PEG_4_-c(RGDfK)]_2_ **13**, [^18^F]AlF-NOTA-E[PEG_4_-c(RGDfK)]_2_ or [^18^F]Alfatide II **14**, [^68^Ga]Ga-NOTA-E[PEG_4_-c(RGDfK)]_2_ **15**, [^18^F]FP-PEG_4_-E[PEG_4_-c(RGDfK)]_2_, **16** (HN-PEG_4_-COOH = 15-amino-4,7,10,13-tetraoxapentadecanoic acid).

The introduction of PEG_4_ (15-amino-4,7,10,13-tetraoxapentadecanoic acid) in the linker was another very popular choice for the synthesis of c(RGD) multimers. A PEG_4_ group was introduced either in between the alpha-amino group of the Glu linker (ligands **11**–**13**) and/or connecting with amide bonds each of the pharmacophore groups (ligands **14**–**16**) (Figure 4). Among those radioligands, the most studied is [^18^F]AlF-NOTA-E[PEG_4_-c(RGDfK)]_2_ or [^18^F]Alfatide II **14**, which is considered an improved version of [^18^F]AlF-NOTA-PEG_3_-E[c(RGDyK)]_2_ or [^18^F]Alfatide I regarding its production process and its stability afterward.

[^18^F]Alfatide II **14** was compared to [^18^F]FDG for parametric monitoring of tumor therapy response to doxorubicin (U87MG tumors) and paclitaxel (MDA-MB-435 tumors) protein-bound particles through a dual tracer imaging approach. The parameters fitted with compartmental modeling from the dual-tracer dynamic imaging were consistent with those from single-tracer imaging, substantiating the feasibility of this methodology. Moreover, dual-tracer imaging was able to measure **14** binding potential value and [^18^F]FDG influx simultaneously to evaluate tumor angiogenesis and metabolism. Such changes are known to precede anatomic changes, offering the promise of early prediction of therapy response with this method [42]. **14** could also monitor early treatment response and predict the therapeutic efficacy of the multi-targeted, anti-angiogenic drug sunitinib in U87MG tumors [43]. Finally, **14** was also evaluated on muscular inflammation [44].

A comparison between the dimeric peptide [^18^F]FP-PEG_4_-E[c(RGDfK)]_2_ (or [^18^F]FP-PRGD_2_) **11**, with [^18^F]FP-PEG_4_-E[PEG_4_-c(RGDfK)]_2_ (or [^18^F]FP-PPRGD_2_) **16**, [45] showed a higher radiolabeling yield for **16** (55 ± 12%) than **11** (25 ± 11%), because of the additional PEG_4_ spacers, which decreased the steric hindrance during the addition of the prosthetic group. In vitro testing (U87MG cells) showed slightly improved avidity for **16** (IC_50_ = 35.8 ± 4.3 nM) in comparison to **11** (IC_50_ = 47.4 ± 5.8 nM). In vivo comparison of **16** and **11** in U87MG human glioblastoma and MDA-MB-435 human melanoma tumor xerographs in mice, also favored **16**, which presented higher U87MG-tumor uptake at all-time points studied (e.g., **16**: 5.32 ± 0.36% ID/g vs. **11**: 3.02 ± 0.44% ID/g, 30 min) and tumor-to-non-tumor background ratios. MicroPET imaging for **16** corresponded to the results of the biodistribution. In addition, micro-PET imaging in the 4T1 murine breast cancer model, which expresses integrin only on the vasculature, confirmed explicit binding of **16** in the tumor vasculature [45].

Guo et al. compared three radioligand dimers bearing [^18^F]AlF-NOTA: [^18^F]AlF-NOTA-E[c(RGDfK)]_2_ **6**, without PEG groups (Figure 3), [^18^F]AlF-NOTA-PEG_4_-E[c(RGDfK)]_2_ **13** with one PEG_4_ between the central glutamic acid and NOTA and [^18^F]AlF-NOTA-E[PEG_4_-c(RGDfk)]_2_ or [^18^F]Alfatide II **14** with two PEG_4_ groups connecting each c(RGD)pharmacophore to the central Glu linker [46]. The study proved the superiority of **14** over the other two. All three multimeric radioligands remained intact after 120 min incubation in mouse serum. Comparison of the cell-binding avidities in U87MG cells with the unlabeled dimer E[c(RGDfK)]_2_ also favored **14** compared to **13** and **6** (E[c(RGDfK)]_2_ > **14** > **13** > **6**). While all radioligands showed a rapid and relatively high uptake in U87MG tumors with satisfactory tumor-to-background ratios, **14** had the highest tumor uptake and the lowest accumulation in the liver [46].

**Figure 5:**
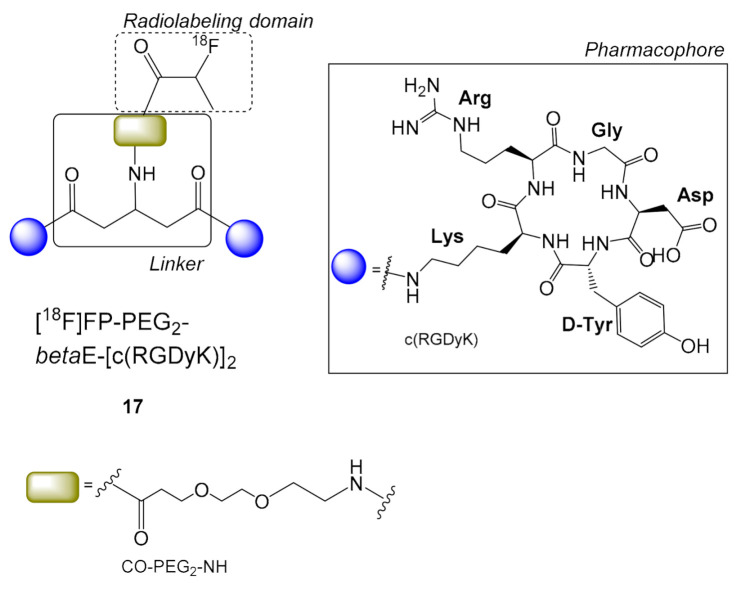
Chemical structure of the symmetric dimer [^18^F]FP-PEG_2_-*β*-E[c(RGDyK)]_2_ **17**. (H_2_N-PEG_2_-COOH = 3-(2-(2-aminoethoxy)ethoxy)propanoic acid).

The effects of a symmetric beta-Glu linker in combination with a PEG_2_ (3-(2-(2-aminoethoxy)ethoxy)propanoic acid) connecting the *β*-glutamate’s amino group with the radiolabeling domain were studied with the ^18^F-labeled c(RGD) homodimeric peptide [^18^F]FP-PEG_2_-*β*-E[c(RGDyK)]_2_ **17** (Figure 5) [47]. The protruding free PEG-amino group of the symmetric *β*-glutamate linker in PEG_2_-*β*-E[c(RGDyK)]_2_ encountered less steric hindrance for the installation of [^18^F]FP compared to the asymmetric PEG-*α*-E[c(RGDyK)]_2_, resulting in a higher-yielding radiolabeling preparation of [^18^F]FP-PEG_2_-*β-*E[c(RGDyK)]_2_ **17** (c.a. 18 ± 3%, synthesis time = 110 min) in comparison with the radioligand [^18^F]FP-E[c(RGDyK)]_2_ **5** (radiochemical yield = 10–15%, synthesis time = 180 min). Biodistribution studies for **17** showed good tumor uptake (30 min p.i.: 3.38 ± 0.23% ID/g; 60 min p.i.: 2.68% ID/g) in A549 mice xenografts, while PET imaging in PC-3 and A549 tumor xenographs showed slightly higher values for PC-3 than A549 (60 min p.i.: 3.38 ± 0.44% ID/g vs. 2.85 ± 0.35% ID/g, respectively). Radioligand **17** was rapidly cleared from the blood by predominately renal excretion and had good stability in vitro and in vivo [47]. However, a direct pharmacokinetic comparison with similar radioligands with asymmetric Glu linkers e.g., **11** was not attempted.

### 2.3. Sugar Amino Acid Linkers on ^18^F Labeled c(RGD) Multimers

**Figure 6:**
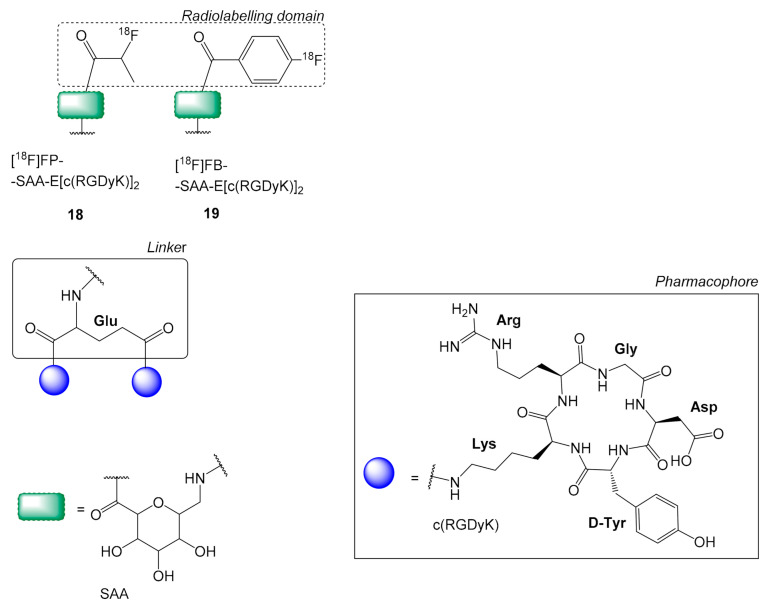
Chemical structures of [^18^F]FP-SAA-E[c(RGDyK)_2_ **18**, [^18^F]FB-SAA-E[c(RGDyK)_2_ **19**, where SAA = 7-amino-l-glyero-l-galacto-2,6-anhydro-7-deoxyheptanamide.

Other linker variations like galactose-based sugar amino acids have been also tested without any significant improvement [48]. In particular, the introduction of a galactose-based sugar amino acid spacer (SAA = 7-amino-L-glyero-L-galacto-2,6-anhydro-7-deoxyheptanamide) in [^18^F]FP-SAA-E[c(RGDyK)_2_ **18**, [^18^F]FB-SAA-E[c(RGDyK)_2_ **19**, (Figure 6) resulted in superior pharmacokinetics than their monomeric analogs, but when **18**–**19** were compared to [^18^F]FP-PEG_3_-E[c(RGDfK)]_2_ **8**, which has a PEG_3_ linker, they all showed a similar pharmacokinetic profile [48]. However, the fact that the radiolabeling yield of **8** was substantially higher (80%) than the yield of **18** (52%) with its laborious four-step synthesis; makes the PEG_3_ linkers a more useful and easily available choice.

### 2.4. The Effect of Linkers on the Stability and Production of ^18^F Labeled Cyclic RGD Multimers

**Figure 7:**
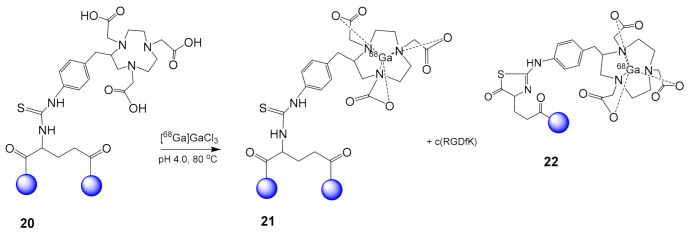
Instability of [^68^Ga]Ga-NOTA-E(c(RGDyK)]_2_ **21** observed during its preparation from **20**.

While [^18^F]Alfatide **10** was already in clinical studies, Lang et al. investigated the stability problems presented during its production [49]. The by-products observed during the production process were due to the neighboring of the glutamic acid to the *α*-amine and thiurea groups, both participating in an intermolecular Lewis acid-catalyzed hydrolysis reaction under acidic conditions. Specifically, during the preparation of [^68^Ga]Ga-NOTA-E(c(RGDyK)]_2_ **21** from **20** under acidic conditions and elevated temperature the nucleophilic addition of thiocarbonyl group of thiourea moiety at the neighboring carbonyl of the glutamic acid leads to the formation of a thiazolidinone ring in **20** and the release of c(RGDfK) (Figure 7) [49]. The introduction of a PEG_3_ spacer in [^18^F]Alfatide I **10** increased the distance between the Glu linker and the thiourea group, reducing this phenomenon. However, when the NOTA chelator was linked to the dimer with a carboxamide bond, it minimized the oxidation of the thiourea motif resulting in a more stable compound: [^18^F]AlF-NOTA-E[PEG_4_-c(RGDfK)]_2_ or [^18^F]Alfatide II **14**. In [^18^F]Alfatide II, the tyrosine of the cyclic RGD part was replaced by a D-phenylalanine, which is not oxidized by heating [50], while the two PEG_4_ groups introduced between each of the c(RGD) motifs and the Glu linker further increased the distance between the pharmacophores, enabling their simultaneous binding to the receptors [50]. It should be mentioned that **14** has also be radiolabeled using the kit formulation method [51,52,53,54].

### 2.5. ^64^Cu Labeled c(RGD) Multimers

The introduction of chelator groups i.e., NOTA, DOTA (1,4,7,10-tetraazadodecane-*N,N,N,N*-tetraacetic acid), facilitates the radiolabeling of c(RGD) multimers with various radionuclides; among them the positron-emitting PET radionuclide ^64^Cu (half-life = 762 min, β^+^ 17.9%, 0.64 MeV), which can be produced from a biomedical cyclotron with various methods [31]. Radiolabeling is achieved by complexation of ^64^Cu with the chelator group in mild conditions.

**Figure 8:**
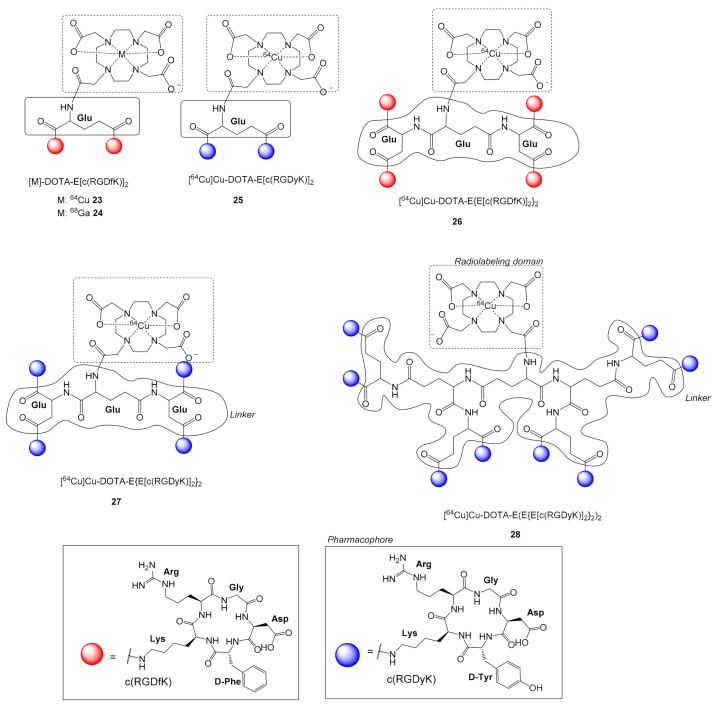
Chemical structures of ^64^Cu-labeled c(RGD) peptides with (Glu)_n_ linkers, [M]-DOTA-E[c(RGDfK)]_2_, where M = ^64^Cu: **23**, M = ^68^Ga: **24**, [^64^Cu]Cu-DOTA-E[c(RGDyK)]_2_ **25** and the c(RGD) tetramers [^64^Cu]Cu-DOTA-E{E[c(RGDfK)]_2_}_2_ **26** and [^64^Cu]Cu-DOTA-E{E[c(RGDyK)]_2_}_2_ **27** and the cRGD octamer [^64^Cu]Cu-DOTA-E(E{E[c(RGDfK)]_2_}_2_)_2_ **28**.

Chen et al. synthesized conjugates of the dimeric c(RGD) peptides E[c(RGDyK)]_2_ and E[c(RGDfK)]_2_ with the DOTA chelator, which were then complexed with ^64^Cu [55]. Both radioligands [^64^Cu]Cu-DOTA-E[c(RGDfK)]_2_ **23** and [^64^Cu]Cu-DOTA-E[c(RGDyK)]_2_ **25** (Figure 8) were used in biodistribution, micro-PET imaging, and whole-body autoradiography studies in athymic female nude mice with MDA-MB-435 breast carcinoma xenografts. Radiotracers **23** and **25** showed specific α_v_β_3_ integrin tumor accumulation (i.e., 60 min p.i.: 3–4% ID/g), with higher retention than previously tested monomeric c(RGD) radioligands. However, activity accumulation of **25** in tumors was significantly higher compared to the d-Phe analog **23**. Liver uptake of the d-Tyr derivative **25** was lower than the d-Phe **23** derivative at early time points, but the difference became marginal with time. Generally, **25** yielded better PET images than **23**. The authors attributed this to the increased hydrophilicity of d-Tyr in **25** compared to d-Phe in **23** [55].

Wu et al. synthesized the tetrameric c(RGD) peptidic radiotracer [^64^Cu]Cu-DOTA-E{E[c(RGDfK)]_2_}_2_ **26** using a scaffold of three glutamic acid residues and compared it with the dimeric analog [^64^Cu]Cu-DOTA-E[c(RGDfK)]_2_ **23** (Figure 8) [56]. The c(RGD) tetramer **26** showed higher integrin-binding avidity (IC_50_ = 16.6 ± 1.3 nM) in comparison to the corresponding dimeric analogue **23** (IC_50_ = 48.4 ± 2.8 nM). Biodistribution and noninvasive microPET studies of tetramer **26** showed rapid, high and specific tumor uptake (U87MG) (30 min p.i.: 9.93 ± 1.05% ID/g; 24h p.i.: 4.56 ± 0.51% ID/g). Ligand **26** showed rapid blood clearance and predominantly renal excretion. The initial high tumor uptake and prolonged tumor retention of the tetramer were attributed to the high integrin avidity and the long blood circulation time, respectively. The latter was due to the increased molecular size. However, the enhanced tumor uptake of the tetramer **26** compared with the dimer **23** was accompanied by a similar increase in renal uptake, while tumor-to-kidney ratios did not increase significantly [56]. Consequently, the therapeutic/diagnostic advantage of a tetramer **26** over a dimer **23** might be modest.

Li et al. used a scaffold of seven glutamic acid residues to synthesize the c(RGD) octamer [^64^Cu]Cu-DOTA]-E(E{E[c(RGDyK)]_2_}_2_)_2_ **28** and compared it with the c(RGD) tetramer [^64^Cu]Cu-DOTA-E{E[c(RGDyK)]_2_}_2_ **27** PET imaging of integrin a_v_β_3_ expressing tumors. [30] The c(RGD) octamer **28** showed significantly higher binding avidity and specificity for integrin a_v_β_3_ (IC_50_ = 10 nM) compared to the tetramer **27** (IC_50_ = 35 nM) (Figure 8). For **27**, the distance between two distant c(RGD) pharmacophores is about 30 bond lengths, which is considered sufficient for simultaneous binding to adjacent integrin a_v_β_3_ receptors, while for the octamer **28** the distance is increased to 40 bond lengths, enabling simultaneous binding with two or more receptors. The octamer **28** showed higher tumor uptake and longer tumor retention compared to the tetramer **27** in both tumor models tested i.e., U87MG, 30 min p.i. **28**: 11.7 ± 0.7% ID/g, **27**: 10.3 ± 1.6% ID/g; c-neu onco-mice 60 min p.i. **28**: 8.9 ± 2.1% ID/g, **27**: 4.4 ± 0.9% ID/g), while the integrin a_v_β_3_ specificity of both tracers was confirmed by successful receptor-blocking experiments. However, a higher uptake and slow clearance in the kidneys was noted for **27**, which was attributed to the integrin positivity of the kidneys, and to its larger molecular size [30]. The above studies indicate that the advantages observed for tetramers and octamers regarding receptor avidity and tumor uptake are counterweighted by their slow renal clearance, which eventually decreases their potential as diagnostic or therapeutic agents.

**Figure 9:**
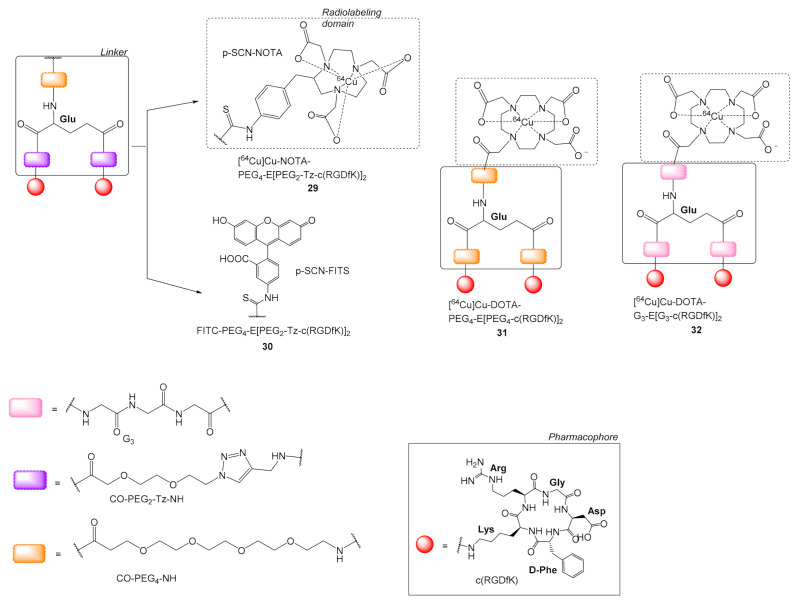
Chemical structures of ^64^Cu and FITC labeled c(RGD) peptides with the Tz-PEG_2_ spacers **29** and **30,** respectively (Tz = triazole group, FITC = Fluorescein isothiocyanate isomer I) and with PEG_4_ **31** and G_3_ **32**.

Hedhli et al. synthesized the dimeric c(RGD) radioligand [^64^Cu]Cu-NOTA-PEG_4_-E[(PEG_2_-Tz-c(RGDfK)]_2_ (Tz = triazole group) **29** (Figure 9) for application in PET imaging and the FITC-PEG_4_-E[(PEG_2_-Tz-c(RGDfK)]_2_ **30** bearing a fluorescent group for in vitro studies [57]. Ligands **29** and **30** are similar to other previously mentioned multimeric c(RGD) peptides bearing PEG groups in the linker region, but they differed in the Tz group, which was formed using CuAAC instead of forming a peptide bond. The binding kinetics against a_v_β_3_ receptor were investigated using surface plasmon resonance. The association K_on_ and dissociation K_off_ constants of **29** and **30** and the commercially available monomeric c(RGDyK) were investigated in immobilized α_V_β_3_ receptors. The unlabeled dimeric peptide NOTA-PEG_4_-E[PEG_2_-Tz-c(RGDfK)]_2_ corresponding to **27**, showed a binding avidity (K_d_ = 0.19 pM), which was approximately 50-fold higher than the binding affinity of the monomeric NOTA-c(RGDfK) (K_d_ = 9.6 pM), while ^64^Cu labeled **29** and FITC labeled **30** showed marginally reduced binding avidity with K_d_ values 1.5 pM and 8.6 pM, respectively. According to the authors, the receptor-bound dimeric c(RGD) peptides **29** and **30** dissociated from α_V_β_3_ at a much slower rate (k_off_ = 2.1 × 10^−6^ s^−1^) compared to the typical adhesive proteins, e.g., the fibrinogen (k_off_ = 9.8 × 10^−4^ s^−1^) and the vibronectin (k_off_ = 2.1 × 10^−4^ s^−1^). In HUVEC cells (human umbilical vein endothelial cells) the binding affinities of **30** (K_d_ = 38.27 nM) and **29** (K_d_ = 33.85 nM) were comparable to the binding affinities of fibrinogen and vibronectin, which are 27 nM and 64 nM, respectively. More importantly, the dissociation constant of the dimeric c(RGD) peptides **29** and **30** was approximately 20-fold lower than most monomeric c(RGD) peptides, and only 2.5-fold higher than the α_V_β_3_ integrin’s antibody LM609 (Kd = 14.4 nM).

Compared to **29**, Shi et al. investigated the replacement of the two PEG_2_-Tz with two PEG_4_ groups in the dimeric ligand **31** [^64^Cu]Cu-DOTA-PEG_4_-E[PEG_4_-c(RGDfK)]_2_ and the replacement of PEG_4_ and the two PEG_2_-Tz with G_3_ groups in dimer **32** [^64^Cu]Cu-DOTA-G_3_-E[G_3_-c(RGDfK)]_2_ and also used a COCH_2_ instead of the C(=S)NHC_6_H_4_CH_2_ moiety connecting the chelator with the spacer-pharmacophore part of the radioligand (Figure 9). [58] The structural alteration marginally increased receptor avidity for **32** (IC_50_ = 62 ± 6 nM), compared to **31** (IC_50_ = 74 ± 3 nM). Dimers **31** and **32** were ^64^Cu-radiolabeled with high yields and specific activity being >50 Ci/mmol. Biodistribution studies showed a very similar kinetic profile for the two radiotracers regarding U87MG tumor uptake and clearance with the PEG_4_-based dimer **31** showing slightly faster blood clearance and lower kidney values [58].

**Figure 10:**
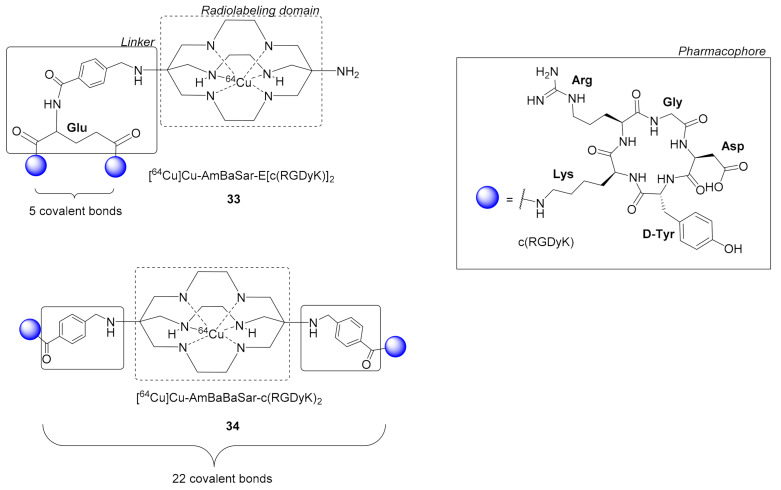
Dimeric c(RGDyK)_2_ radioligands [^64^Cu]Cu-AmBaSar-E[c(RGDyK)]_2_ **32** and [^64^Cu]Cu-AmBaBaSar-c(RGDyK)_2_ **33** bearing the hexaazamacrobicyclic sarcophagine (Sar) chelator, AmBa = 4-(Aminomethyl)benzoic acid.

Liu et al. synthesized two dimers [^64^Cu]Cu-AmBaSar-E[c(RGDyK)]_2_ **33** and [^64^Cu]Cu-AmBaBaSar-c(RGDyK)_2_ **34** using the cage hexaazamacrobicyclic sarcophagine (Sar) chelator, for labeling with ^64^Cu under mild conditions, in combination with the linker AmBa (AmBa = 4-(Aminomethyl)benzoic acid). Ligands were formed by reacting 4-bromomethylbenzoic acid with one **33** or both **34** amine groups of Sar protruding the cage cavity (Figure 10). After radiolabeling, the dimers were further evaluated in vitro and in vivo [59,60]. Radioligands **33** and **34** were proved very stable (intact tracer > 95% during HPLC analysis, 60 min after injection), both in vitro and in vivo and this was attributed to the cross bridged and cage-like configuration of the Sar chelator. Dimeric radioligand **33** showed higher tumor uptake than its respective monomeric, (20 h p.i. **33**: 1.76 ± 0.38% ID/g, [^64^Cu]Cu-AmBaSar-c(RGD): 0.65 ± 0.05% ID/g) and generally more favorable pharmacokinetics, due to the polyvalency effect [59]. However, the bi-functionalized Sar dimeric ligand **34** was proved superior to **33** both in vitro, by displaying higher avidity, i.e., **34**: IC_50_ = 6.0 ± 0.9 nM, **33**: IC_50_ = 10.0 ± 0.5 nM, and in vivo, by showing higher tumor uptake i.e., 1h p.i **33**: 3.04 ± 0.25% ID/g, **34**: 6.16 ± 0.88% ID/g. The difference according to the authors was due to the distance between the two pharmacophores, which is 5 bonds (Glu linker) in the case of **33,** while it is 22 covalent bonds in **34** due to the intervention of the AmBaBaSar group (Figure 10). Thus, in **33** the simultaneous binding on two integrin receptors was less likely compared to **34**, where the increased distance and flexibility permit such binding interactions [59,60].

### 2.6. ^68^Ga-Labeled RGD Multimers

An interesting alternative for the ^18^F and ^64^Cu cyclotron-produced PET radionuclides is the generator-produced ^68^Ga, which can be eluted from an inhouse ^68^Ge/^68^Ga generator (^68^Ge, T_1/2_ = 270.8 days) and has optimal physical characteristics (β^+^ 89%, 1.92 MeV) for PET imaging. Furthermore, its half-life of 68 min, is compatible with the pharmacokinetics of many peptides [31]. The following section refers to the ^68^Ga radiolabeled c(RGD) multimers in comparison to their ^18^F and ^64^Cu structural analogs, as well as to some ^68^Ga radiolabeled c(RGD) multimers not previously mentioned.

Siitonen et al. prepared the [^68^Ga]Ga-DOTA-E[c(RGDfK)]_2_ **23** (Figure 8), which was then used for PET imaging of Shank-associated RH domain-interacting protein (SHARPIN)-Regulated Integrin Activity in mice. [61] SHARPIN is a cytosolic protein that plays a key role in the activation of nuclear factor κ-light-chain enhancer of activated B cells and regulation of inflammation. Furthermore, SHARPIN controls integrin-dependent cell adhesion and migration in several normal and malignant cell types. Loss of SHARPIN correlates with increased integrin activity in mice. Increased integrin activity due to loss of SHARPIN protein would affect the uptake of the α_v_β_3_-selective **23**, both in several tissue types and in the tumor microenvironment. PET imaging in vivo was evaluated in wild-type (wt) and SHARPIN-deficient mice (Sharpin^cpdm^, where cpdm 5 designates chronic proliferative dermatitis in mice) with and without melanoma tumor allografts. Sharpin^cpdm^ mice with a spontaneous null mutation in the Sharpin gene and their wt littermates with or without B16-F10-luc melanoma tumors were studied using in vivo PET/CT imaging and ex vivo measurements with **23**. The ex vivo uptake of **23** in the mouse skin and tumor was significantly higher in Sharpin^cpdm^ mice than in wt mice, while B16-F10-luc tumors were detected 4 d after inoculation, without differences in volume or blood flow between the mouse strains. PET imaging even after 10 days of inoculation revealed significantly higher uptake in the tumors transplanted into Sharpin^cpdm^ mice than in wt mice, while tumor vascularization was also increased in the Sharpin^cpdm^ mice, indicating that SHARPIN may also have important regulatory roles in controlling the tumor microenvironment.

**Figure 11:**
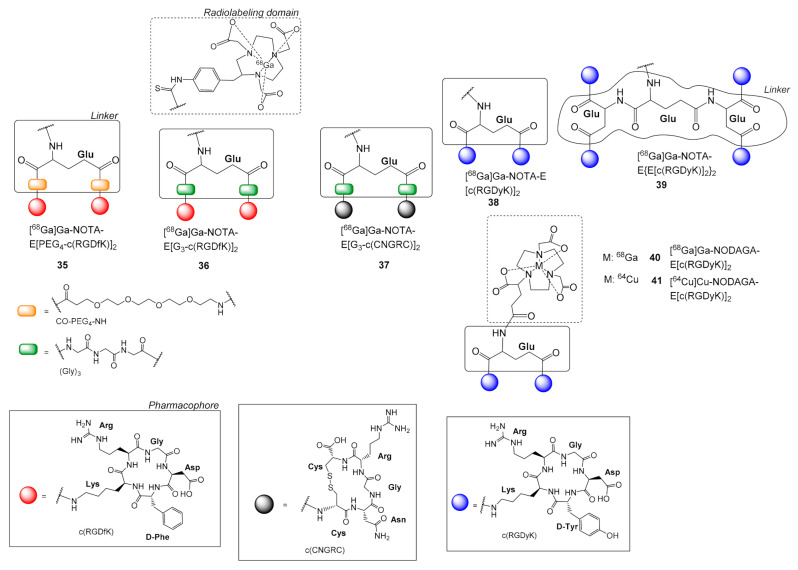
Chemical structures of the dimeric radioligands [^68^Ga]Ga-NOTA-E[PEG_4_-c(RGDfK)]_2_ **35**, [^68^Ga]Ga-NOTA-E[G_3_-c(RGDfK)]_2_ **36** and [^68^Ga]Ga-NOTA-E[G_3_-c(CNGRC)]_2_ **37**, [^68^Ga]Ga-NOTA-E[c(RGDyK)]_2_ **38**, [^68^Ga]Ga-NOTA-E{E[c(RGDyK)]_2_}_2_ **39**, [^68^Ga]Ga-NODAGA-E[c(RGDyK)]_2_ **40** and [^64^Cu]Ga-NODAGA-E[c(RGDyK)]_2_ **41.**

Liu et al. studied the two dimeric c(RGD) pharmacophores E[PEG_4_-c(RGDfK)]_2_ **35** and E[G_3_-c(RGDfK)]_2_ **36** linked with [^68^Ga]Ga-NOTA complex (*p*-SCN-Bn-NOTA) (Figure 11) [62]. The ^68^Ga analogs **35** or **36**, contained the groups PEG_4_ and G_3_ linkers, respectively, only between the Glu scaffold and the c(RGD) pharmacophores, while in their [^64^Cu]Cu-DOTA analogs **31**, **32** (Figure 9) [58] a PEG_4_ or G_3_ linker is additionally included between the chelator group and the dimeric c(RGD) ligands. The presence of the additional linker PEG_4_ or G_3_ in between the chelator group and the c(RGD) pharmacophores dramatically improved α_v_β_3_ integrin receptor avidity, i.e., NOTA-E[c(RGDfK)]_2_ IC_50_ = 100.04 ± 2.85 nM, NOTA-E[PEG_4_-c(RGDfK)]_2_ **35** IC_50_ = 33.96 ± 2.17 nM, NOTA-E[G_3_-c(RGDfK)]_2_ **36** IC_50_ = 66.38 ± 3.75 nM [62]. These values are in the same range as their DOTA analogs (**32** IC_50_ = 62 ± 6 nM, **31** IC_50_ = 74 ± 3 nM), [58] indicating that the chelator did not significantly affect avidity. Instead, binding avidity was seriously affected by the applied spacer group. The benefits of using a PEG_4_ or G_3_ in the spacer were also observed in the biodistribution experiments, where it was shown that both radiotracers had higher (U87MG and MDA-MB-435) tumor uptake than the reference NOTA-E[cRGDfK)]_2_ [63], while again, small differences were observed in comparison to their DOTA counterparts [58].

The imaging properties of the dimeric c(RGD) and c(NGR) radioligands [^68^Ga]Ga-NOTA-E[G_3_-c(RGDfK)]_2_ **36** and [^68^Ga]Ga-NOTA-E[G_3_-c(CNGRC)]_2_ **37**, respectively, against angiogenesis were compared by Shao et al. [64] NGR peptides identified from a phage display are known to target the aminopeptidase N (APN/CD13) receptor, which has multiple functions associated with the progression of malignancy such as angiogenesis [65]. The two ligands presented similar pharmacokinetic profile, stability and tumor uptake (HT1080 fibrosarcoma) i.e., 60 min p.i., **36**: 6.89 ± 2.34% ID/g; **37**: 5.18 ± 1.06% ID/g [64].

Li et al. also tested a dimeric [^68^G]Ga-NOTA-E[c(RGDyK)]_2_ **38** and tetrameric [^68^Ga]Ga-NOTA-E{E[c(RGDyK)]_2_}_2_ **39** analogue of c(RGD) for integrin α_v_β_3_ targeting [66]. The tetramer **39** was proved superior in vitro in U87MG cells, **39** IC_50_ = 16.1 ± 3.1 nM > **38** IC_50_ = 60.1 ± 7.6 nM > monomer IC_50_ = 218 ± 28 nM, while quantitative microPET imaging studies showed that it also had the highest tumor uptake but in combination with the highest kidney accumulation. Thus, the dimeric structure was again the most favorable choice of this study.

Oxboel et al. prepared the complexes of the dimeric pharmacophore NODAGA-E[c(RGDyK)]_2_ with ^68^Ga and ^64^Cu, **40** and **41** respectively (NODAGA = 1,4,7-triazacyclononane-1-glutaric acid-4,7-diacetic acid), to evaluate them as angiogenesis PET tracers (Figure 9). Radioligand [^68^Ga]Ga-NODAGA-E[c(RGDyK)]_2_ **40**, [^64^Cu]Cu-NODAGA-E[c(RGDyK)]_2_ **41** were tested in nude mice bearing either human glioblastoma (U87MG) or human neuroendocrine (H727) xenograft tumors [67]. PET/CT scans were conducted at selected time points and used for calculating the tracer uptake in tumors (% ID/g) in parallel with biodistribution studies. Both tracers **40**, **41** showed similar uptake in xenograft tumors 60 min after injection, U87MG, **40**: 2.23 ± 0.08% ID/g, **41**: 2.31 ± 0.15% ID/g; H727, **40**: 1.53 ± 0.06% ID/g vs. **41**: 1.48 ± 0.08% ID/g. Biodistribution studies showed similar tracer uptake for **40** and **41**, however, **40** showed a slightly more stable tumor retention [67].

**Figure 12:**
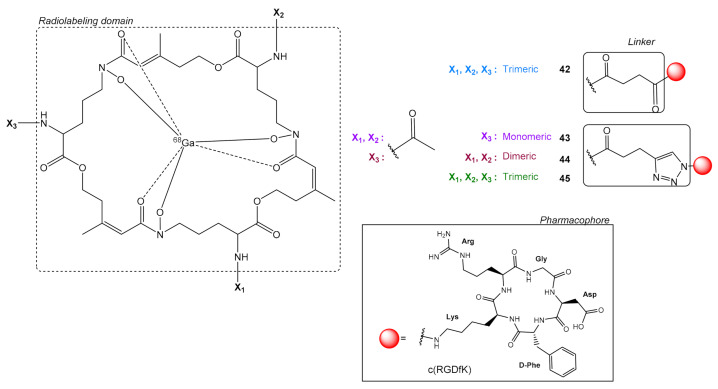
Chemical structures of conjugates between the natural chelator fusarinine C (FSC) and c(RGDfK) pharmacophores, [^68^Ga]Ga-FSC-[E-c(RGDfK)]_3_ **42**, [^68^Ga]Ga-FSC-(CH_2_)-Tz-c(RGDfK) **43**, [^68^Ga]Ga-FSC-[(CH_2_)-Tz-c(RGDfK)]_2_ **44**, [^68^Ga]Ga-FSC-[(CH_2_)-Tz-c(RGDfK)]_3_ **45.**

The cyclic peptide siderophore (FSC) (Figure 12), which has very good complexing properties for ^68^Ga has been used as a scaffold for the synthesis of polymeric c(RGD) ligands. [68] Multimeric c(RGD) radioligands can be prepared either through the peptidic bond formation by coupling the c(RGD) peptide to the Fe-complex of the deacetylated form of Fusarinine-C (FSC) [68] or through a triazole ring formation using click CuAAC chemistry [23]. Subsequent Fe-demetallation (with Na_2_EDTA) allows radiolabeling with ^68^Ga.

In this context, Knetsch et al. [68] prepared the trimeric c(RGD) peptide [^68^Ga]Ga-FSC-[E-c(RGDfK)]_3_ **42** and tested it in vitro in α_v_β_3_ positive human melanoma M21 cells (vs. control integrin negative M21-L) [68]. Ligand **42** showed high avidity (IC_50_ = 1.8 ± 0.6 nM) and receptor-specific internalization, while in vivo it showed specific tumor uptake (60 min p.i., M21: 4.25 ± 0.64% ID/g, M21-L: 1.13 ± 0.38% ID/g) with good contrast ratios i.e., tumor/blood = 8.2, tumor/muscle = 7.4. The reference monomer [^68^Ga]Ga-NODAGA-c(RGD) on the other hand was inferior tumor/blood: 11.3, tumor/muscle: 6.1. Trimeric **42** was mainly excreted via the kidneys showing higher accumulation than the reference monomer, i.e., 60 min p.i. Kidneys: 4.7 ± 0.5% ID/g vs 1.5% ID/g, respectively. However, only a one-time point was investigated (60 min p.i.) and not a complete kinetic analysis [68].

In a later study, Kaeopookum et al. prepared the monomeric, dimeric, and trimeric c(RGD) radioligands: [^68^Ga]Ga-FSC-(CH_2_)-Tz-c(RGDfK) **43**, [^68^Ga]Ga-FSC-[(CH_2_)-Tz-c(RGDfK)]_2_ **44**, [^68^Ga]Ga-FSC-[(CH_2_)-Tz-c(RGDfK)]_2_ **45**, respectively. Their binding properties for integrin α_v_β_3_ were evaluated in vitro as well as in vivo and compared with the monomeric [^68^Ga]Ga-NODAGA-c(RGDfK) and trimeric [^68^Ga]Ga-FSC-[suc-c(RGDfK)]_3_ [23]. All ^68^Ga-labeled c(RGDfK) peptides displayed fair hydrophilicity (logD = −2.96 to −3.80), low protein binding, and were stable in phosphate buffered-saline and serum up to 2 h. In vitro receptor binding avidity and internalization assays in M21 cells showed specific uptake of all derivatives, which increased with the number of c(RGD) motifs i.e., **45** > **44** > **43**. However, these in vitro avidity values did not exactly correspond with the in vivo (U87MG xenographs in mice) results, the monomer had the lowest tumor, but the dimer showed higher tumor uptake compared to the trimer i.e., 90 min p.i., **44**: 8.19 ± 0.41% ID/g > **45**: 3.98 ± 0.64% ID/g > **43**: 2.73 ± 0.28% ID/g, while tumor uptake for the reference trimeric [^68^Ga]Ga-FSC-(suc-c(RGDfK))_3_ was 4.95 ± 1.10% ID/g. The dimeric **44** also showed the best tumor-to-background ratios. All radiolabeled compounds showed fast blood clearance and high accumulation in kidneys. The authors correlated the high tumor uptake of dimeric **44** compared to the trimeric **45** to the lower density of α_v_β_3_-integrins on U87MG cells in comparison with the human melanoma M21 cells (preventing simultaneous binding) and this explanation was partly confirmed by the in vitro binding results [23].

**Figure 13:**
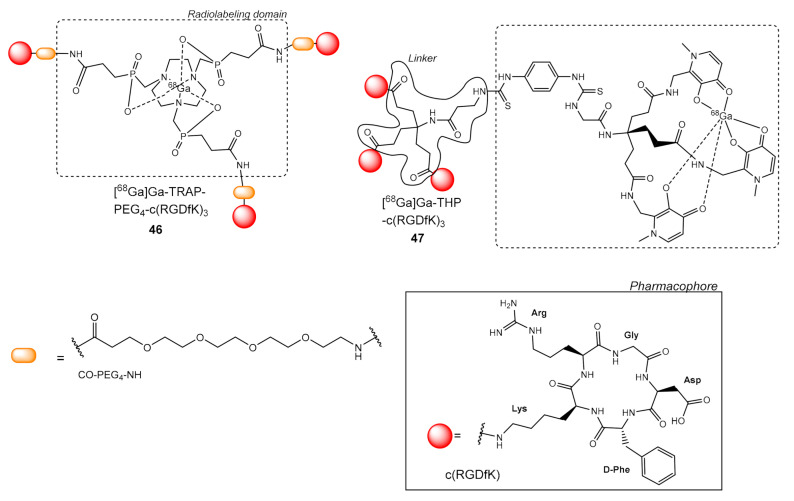
Chemical structures of ^68^Ga trimers of c(RGDfK) with TRAP **46** and THP **47** chelator groups.

A very interesting chelator group initially used by Notni et al. for c(RGDfK) multimerization is TRAP (1,4,7-triazacy-clononane-1,4,7-tris[(2-carboxyethyl)methylenephosphinic acid]) [69]. TRAP allows the efficient and high yielding complexation of ^68^Ga^3+^, while presenting multiple sites for conjugation of pharmacophores. (Figure 13) Notni et al. did an extensive work testing various linkers i.e., PEG_4_, PEG_8_, Ahx (6-aminohexanoic acid), Glu, -CH_2_-Tz-Ahx, in between TRAP and c(RGDfK) to conclude in the choice of PEG_4_ and ligand **46**, which showed the higher avidity for integrin α_v_β_3_ (M21/negative M21-L). **46** showed in vitro a 7-fold higher avidity compared to the monomers F-Galacto-RGD and Ga-NODAGA-c(RGDyK), and in vivo high tumor uptake (60 min p.i., 6.08 ± 0.63% ID/g), was and fast renal clearance.

In a study by Lobeek et al., they compared dimeric **24** and trimeric c(RGDfK) ligands **42**, **46**, and **47**, which contains a bifunctional tris(hydroxypyridinone) chelator THP (H_3_THP-Ph-NCS) (Figure 13). During the in vitro experiments, the dimeric ligand **24** was superior; presenting the lowest IC_50_ value (3.8 ± 0.7 nM), while IC_50_ values of the trimeric structures did not significantly differ (9.0–11.4 nM). The FSC analog **42** presented the highest tumor uptake in the SK-RC- 52 (human renal cell carcinoma) model (60 min p.i. 12.5 ± 2.5% ID/g), while the rest of the ligands had lower values (all in the 4.4–5.3% ID/g). In the FaDu model (human squamous cell carcinoma, tumor cells expressing α_v_β_3_ integrin solely on the neovasculature; α_v_β_3_ integrin–negative tumors), **42** was significantly higher (60 min p.i. 1.9 ± 0.3% ID/g) than that of **46** (1.0 ± 0.2% ID/g), but it did not significantly differ from the other two ligands **24** (1.6 ± 0.5% ID/g) or **47** (2.2 ± 0.7% ID/g). The optimal choice according to the authors were the trimeric structures **42** and **47** [70].

The studies so far have shown that there is no significant advantage in using radiolabeled tetramers: E{E[c(RGD-X-K)]_2_}_2_ (X = f or y) over their dimeric analogs: E[c(RGD-X-K)]_2_ (X = f and y), regarding tumor to background (T/B) ratios or normal organ uptake. It seems that there is a limit on the benefits provided by the increasing pharmacophore multiplicity since over two c(RGD) pharmacophores increased the uptake in normal organs (kidneys, liver, lungs, and spleen). In addition, multiplicity increases production complexity and costs; two factors, which act prohibitively for the future development of multimers c(RGD)_n_ with *n* > 4, as integrin α_v_β_3_-targeting radiotracers [20].

### 2.7. Clinically Applied RGD Multimers

A number of multimeric integrin-targeting c(RGD) radioligands have been applied since 2014 for prospective human studies, mainly focusing on oncological diseases (Table 1) [24,25,71]. Ligand [^18^F]FP-PRGD_2_ **8** was the first dimeric c(RGD) tracer that was clinically applied in healthy volunteers. Mitra et al. showed that this tracer had good tolerance as well as favorable biodistribution and dosimetric characteristics [72], leading to its FDA approval as an exploratory investigative new drug (IND 104150) in human subjects. Iagaru et al. reported that in a pilot evaluation of radioligand **8**, eight women with newly diagnosed or recurrent breast cancer (BCa) underwent PET/CT with **8** and the commonly used radiopharmaceutical in PET imaging [^18^F]FDG [73]. The radioligand **8** showed high and specific uptake in primary cancer, as well as in the metastatic lesions, with no safety issues reported or measured. The biodistribution of **8** in cancer patients was extensively investigated in a later study by Minamimoto et al. reporting high tracer accumulation in the bladder and kidneys, due to the tracer’s predominant renal clearance, followed by the choroid plexus, spleen, salivary glands, thyroid, liver, pancreas, and bowel [74]. The above results in addition to the observed good tumor-to-background ratios suggested the ligand’s **8** suitability for further clinical use. Furthermore, the dimeric c(RGD) **8** was found clinically superior to the monomers [^18^F]FP-galacto-E(c(RGDfK)] ([^18^F]FP-galacto-RGD) and [^18^F]FP-E-c(RGDfK) ([^18^F]Fluciclatide) [24,75], which was in accordance with the preclinical animal studies [48]. Additionally, the lack of significant correlation between tumor uptake for **8** and [^18^F]FDG confirmed that the two PET tracers provide different molecular information [74].

In a clinical study investigating **10**’s feasibility for lung cancer detection, 26 patients with suspected lung cancer underwent PET/CT with this tracer before surgery and puncture biopsy. Standardized uptake values (SUVs) and tumor-to-blood ratios were measured, and diagnoses were also pathologically confirmed. Results showed that **10** was able to clearly identify all primary lesions with desirable image contrast (sensitivity = 100%, specificity = 44%, accuracy = 81%, positive predictive value [PPV] = 77% and negative predictive value [NPV] = 100%). The SUV for malignant lesions was significantly higher than that for hamartomas. However, it was difficult to clearly differentiate inflammatory or inflammatory pseudotumors from malignant lesions [76].

In another study of **10**, 13 patients with non-small cell lung cancer (NSCLC) underwent PET/CT before surgery [77]. All malignant lymph nodes (LNs) were successfully visualized with a sensitivity of 100%, a specificity of 95%, and an accuracy of 95%. SUVmax, SUVmean, and SUV ratios in malignant LNs were significantly higher than in benign LNs. Similar results were observed in patients with adenocarcinoma and squamous cell carcinoma. Tracer **10** showed high sensitivity (83.9–100%), specificity (78.6–96.7%), and accuracy (81.7–96.9%) according to thresholds calculated from receiver operating characteristic curves.

Hitherto, the majority of clinical studies regarding c(RGD) multimers have been conducted for tracer [^18^F]Alfatide II **14** [52,53,54]. The radiotracer was initially (2015) investigated in five healthy volunteers and nine patients with brain metastases (identified by MRI and/or CT) originating from various primary tumors i.e., lung, ovarian, gastric [54]. **14** was well tolerated without any serious tracer-related adverse events. The tracer showed rapid clearance from the blood pool and kidneys, while the organs with the highest absorbed dose were the kidneys and the spleen. Further, the detection rate of **14** was compared to other imaging modalities, in particular, CT and [^18^F]FDG PET/CT. The comparison revealed that all 20 brain lesions were visualized by **14**, while 13/20 lesions were visualized by CT and only 10/20 by [^18^F]FDG PET/CT [54]. Of note, however, is the fact that [^18^F]FDG is not an optimal imaging biomarker for brain tumors; instead, amino acid PET tracers, such as l-[methyl-^11^C]methionine (^11^C-MET), *O*-(2-[^18^F]fluoroethyl)-l-tyrosine ([^18^F]FET), and 3,4-dihydroxy-6-[^18^F]Fluoro-l-phenylalanine ([^18^F]FDOPA), yield better results regarding brain tumors’ detection [80,81]. **14** was also investigated for the diagnosis of bone cancer metastasis (2015) in 11 patients (*n* = 7 lung cancer, *n* = 2 cancer of unknown primary site, *n* = 1 gastric cancer, *n* = 1 urinary bladder cancer associated with gastric cancer) who underwent PET/CT with [^18^F]FDG and **14** [53]. The final diagnosis of bone lesions was established based on the comprehensive assessment of all available data and clinical follow-up. Bone metastases were divided into four groups: osteolytic, osteoblastic, mixed, and bone marrow. PET/CT imaging using **14** detected the bone metastatic lesions with good contrast and higher sensitivity (positive rate of 92%) than [^18^F]FDG (77%), especially in detecting osteoblastic (70% vs. 53%) and bone marrow metastatic lesions (98% vs. 77%). PET/CT sensitivity of **14** in osteolytic metastasis was 100%, while for [^18^F]FDG was 90% [53]. Moreover, **14** was compared to [^18^F]FDG for detecting BCa, in a cohort of 44 female patients [52]. PET/CT image analysis was based on visual and semi-quantitative analysis (SUV_max_, SUV_mean_). In total, 42 BCa lesions and 11 benign breast lesions were confirmed by histopathology. Both **14** and [^18^F]FDG showed higher uptake for BCa lesions than benign ones (*p* < 0.05) with **14** showing less uptake and area under the curve than [^18^F]FDG. Both **14** and [^18^F]FDG had high sensitivity (88.1% vs. 90.5%), high positive predictive value (PPV 88.1% vs. 88.4%), moderate specificity (54.5% vs. 54.5%), and moderate negative predictive value (NPV 54.5% vs. 60.0%) for differentiating BCa from benign breast lesions. Overall, **14** showed a diagnostic value comparable to that of [^18^F]FDG but was not superior in the identification of BCa. The combination of **14** and [^18^F]FDG, increased sensitivity to 97.6% and NPV to 85.7%, while the PPV was slightly increased to 89.1%, without any change in specificity (54.5%) [52].

Additionally, when this ligand NOTA-E[PEG_4_-c(RGDfK)]_2_ was labeled with ^68^Ga, **15** ([^68^Ga]Ga-RGD_2_) was compared to [^18^F]FDG in LCa patients. [79] Thirty-one patients with pathologically confirmed tumors were enrolled, (21 NSCLC, and 10 small cell lung, SCLC). PET/CT images were acquired using **15** and [^18^F]FDG. The SUVs for [^18^F]FDG (SUV_max_, SUV_mean_) were not significantly different between NSCLC and SCLC patients. On the contrary, **15** uptake of SCLC patients was at background levels and significantly lower than that of NSCLC patients, indicating lower α_v_β_3_ targeting level for c(RGD) in SCLC. The dimeric ligand **15**, could not only detect but also differentiating NSCLC and SCLC cases while detecting intra-tumor heterogeneities [79]. Tracer **15** was also used for differentiating NSCL and tuberculosis (21 NSCLC patients and 13 TB patients were recruited). The values noted for **15** regarding SUV_max_ and SUV_mean_ and area under the curve were significantly different between NSCLC and TB, while the visual differentiation diagnostic specificity of **15** was higher than that of [^18^F]FDG (84.62% vs. 53.85%), with one-third of false-positive rate (15.4%/46.2%) over the [^18^F]FDG rate. In addition, for the detection of NSCLC lymph nodes, **15** showed superior specificity (100% vs. 66.7%), [^18^F]FDG (87.5% vs. 75%) [51].

The dimeric structure **12** (Figure 3) was investigated in comparison to [^18^F]FDG in 91 LCa patients [78]. Tracer **12** was well tolerated, while it rapidly cleared from the blood pool, mainly through the urinary system. The SUVs for proven malignancies were significantly higher than benign lesions with **12** showing a sensitivity of 84%, specificity of 91%, and accuracy of 86%, exhibiting a diagnostic value comparable to [^18^F]FDG for LCa detection. Moreover, **12** was more specific than [^18^F]FDG PET/CT in assessing LN metastasis, with PPV of 90% (30% for [^18^F]FDG) and NPV and 94% (91% for [^18^F]FDG), respectively [78].

Although being structurally different, all the clinically investigated RGD peptides, including monomers and dimers, depict very similar in vivo pharmacokinetic properties [24]. Regarding **8**, and **14**, the two most clinically studied dimers, they were able to detect integrin-positive tumors with good imaging contrast, while showing comparable imaging properties and pharmacokinetics, and while exhibiting high sensitivity (for primary lesions 83.3–100% and for metastatic lesions 70–100%) and specificity for tumor detection and staging. Besides the urogenital system, moderate to prominent off-target uptake was observed for both in the liver, which can be an issue for detecting hepatic tumors or metastases [43,52,82].

Until recently, only a small number of clinical investigations of dimeric RGD peptides have been reported, and the sensitivity/specificity between dimers and monomers has not been compared in the same patients. Thus, additional evaluation with large cohorts is needed to determine if the multimeric strategy provides higher sensitivity and specificity for tumor detection and staging than the monomeric RGD compounds and the golden standard [^18^F]FDG [24].

## 3. Discussion

Multimeric PET radioligands consist of identical binding motifs (pharmacophores) connected to a single backbone (linker) attached to a group, which can be labeled with a positron-emitting radionuclide suitable for PET molecular imaging (radiolabeled domain). Among the various PET multimeric radioligands investigated for targets like integrin α_v_β_3_, PSMA, GRPr, VEGFR, and EGFR-TKI, the ones targeting integrin α_v_β_3_ are the most studied and the only category which has reached the clinical stage of development (Table 1). Multimeric c(RGD) analogs are a fine example, providing proof that multimerization can improve ligands characteristics like receptor avidity and tumor uptake.

Several factors regarding the design of c(RGD) multimeric radioligands should be taken under consideration. One of the most important factors is the length and flexibility of the linker (1) connecting the chelator group with the multimeric scaffold and (2) connecting the various pharmacophores. Regarding the first case of linker (1), several examples of ^18^F-labeled compounds, **7**–**10** (Figure 3) have shown that the introduction of PEG group in-between the pharmacophores and the labeling site not only improves the overall radiolabeling yield but also reduces the renal uptake and increases tumor-targeting efficacy [36]. Specifically, the introduction of PEG_3_ minimized the instability factors observed in the acidic and high-temperature radiolabeling conditions of **10**, due to the thiourea linkage of the labeling site with the α-amine of the Glu linker [50]. Considering the second case (2), the length of the linker defines the distance between the two pharmacophores. Cyclic RGD dimeric peptides, where the Glu linker was connected with each c(RGD) using additional groups like PEG_4_ or G_3_ e.g., **14**–**16** (Figure 4) or **29**–**32** (Figure 9) have shown better results, with respect to tumor uptake, than other ligands with shorter linkers [20]. In particular, for c(RGD) dimers, it seems that this distance has to be in the range of 30–38 bonds like in **26**, **27** [30] or the SAR conjugate **33** [57,58] for achieving bivalency, and eventually leading to higher integrin α_v_β_3_ binding avidity [20].

Several pharmacodynamic models have been proposed as an explanation for the observed improvements in binding avidity, reduced receptor off-rate, which eventually result in high tumor uptake. One of the models suggests simultaneous binding of the ligand with two receptors on the cell surface; this can be accomplished with the utilization of extremely long spacers, which cover the distance between two receptors on the cell surface. However, extremely long spacers do not always prove to be advantageous for the pharmacodynamic or pharmacokinetic ligand characteristics, because they may prevent other actions such as the internalization of the ligand or may worsen its pharmacokinetic properties, resulting in reduced tumor uptake. Another model describing the improved effects observed for multimeric ligands is based on the improved statistical effect, in this case, the ligand binds to one receptor, but its apparent local concentration of the ligand in proximity to the receptors is increased. This seems to be the most likely explanation for ligands with short linkers [1].

Another factor regarding the design of c(RGD) multimers is the number of c(RGD) pharmacophores included. Increasing the number of pharmacophores had a positive effect on ligand avidity and cell binding in vitro. Nevertheless, this effect did not always correspond with similar advantages in vivo, since increasing peptide multiplicity, in many cases resulted in a parallel increase of ligand uptake in normal organs. Thus, the benefits of multiplicity seem to have limits. Among the multimers summarized in this article, the dimers seem the most successful cases and that is also the reason they have further advanced in the clinic, over the other tracers.

Several examples of c(RGD) multimers for PET imaging of integrin α_v_β_3_ have been studied so far, which have been labeled with various PET radionuclides i.e., ^18^F, ^68^Ga, and ^64^Cu (for a list of compounds included in the article along with the α_v_β_3_ expressing cells/tumor models tested refer to Table 2). The ^18^F analogs and specifically dimeric [^18^F]F-c(RGD)_2_ ligands have been the most successful and have already reached the stage of clinical development for various applications utilizing integrin α_v_β_3_ imaging like cancer (ligands **8**, **10,** and **14**). However, the NOTA analogs, which are labeled using [^18^F]AlF, a much easier, faster, and high yielding production procedure (40 min and 20 min for kit radiolabeling, yield 42%, radiochemical purity > 95%), have significant advantages for their future clinical application, especially after the introduction of kit formulations. Among the two compounds developed so far: [^18^F]Alfatide I **10** and [^18^F]Alfatide II **14**; the second is considered more stable regarding a possible intermolecular Lewis acid-catalyzed hydrolysis during its production. Besides ^18^F, another popular positron-emitting radioisotope is ^68^Ga, which can be produced by a ^68^Ge/^68^Ga generator. So far one clinical study has been published for each of the ^68^Ga labeled dimeric structures, **12** and **15**. Tracer **12** showed similar pharmacokinetics with **10**, which can also be labeled with ^68^Ga, and is of alike chemical structure to **14**. Consequently, additional results of clinical studies regarding [^68^Ga]Ga-c(RGD)_2_ dimers i.e., **12** or **15** are expected to be published in the near future.

Finally, regarding the advantages of multimeric c(RGD) analogs in clinical studies, according to the limited data generated so far, the dimeric structures seem to have an advantage over their monomeric analogs. Even so, additional evaluation is needed with large cohorts of patients in order to determine if the multimeric strategy provides higher sensitivity and specificity for tumor detection and staging over the monomeric RGD compounds and the golden standard [^18^F]FDG.

## 4. Concluding Remarks

This article describes multimeric c(RGD) ligands targeting integrin α_v_β_3_ receptors for PET molecular imaging of tumors. Multimerization is generally advantageous and multimeric c(RGD) radioligands have increased binding avidity against α_v_β_3_ receptors and are more effective in tumor targeting compared to monomeric c(RDG) radioligands. However, when the number of c(RGD) pharmacophores was increased above 2, it did not enhance the pharmacokinetic properties of the ligand in vivo, despite the benefits of multimerization observed in vitro. The length and flexibility of the linker connecting the c(RGD) pharmacophores and the linker connecting the multimerization scaffold with the chelator group have a significant role in the biological activity of the multimeric c(RGD) ligands. Clinical studies are expected to bring forward valuable information regarding the application benefits of multimeric c(RGD) ligands.

## Figures and Tables

**Table 1:** Clinical studies with multimeric c(RGD) molecules.

Imaging Agent	Year	# Patients	Confirmation	Neoplasm	Ref.
[^18^F]FP-PRGD_2_ PET/C **8**	2014	8	HP	BCa	[73]
[^18^F]Alfatide I **10**	2015	26/16	HP	LCa/Lnd	[76]
[^18^F]Alfatide I PET/CT **10**	2017	13	HP	Lnd	[77]
[^18^F]Alfatide II PET/CT **14**	2015	5 (HV) 9	MRI/CT	BrCa	[54]
[^18^F]Alfatide II PET/CT **14**	2015	30		BnCa	[53]
[^18^F]Alfatide II PET/CT **14**	2018	44	HP	BCa	[52]
[^68^Ga]Ga-NOTA-PRGD_2_ PET/CT **12**	2015	91 159	HP	Lnd	[78]
[^68^Ga]Ga-RGD_2_ PET/CT **15**	2017	31 (21/10)	HP	NSCLC/SCLC	[79]
[^68^Ga]Ga-RGD_2_ PET/CT **15**	2016	21/13	HP	NSCLC/TB	[51]

Abbreviations used: HP: Histopathology; Lnd: Lymph nodes; BCa: breast cancer; LCa: lung cancer; BrCa: Brain cancer; BnCa: Bone Cancer; NSCLC: non-small cell lung cancer; SCLC: small cell lung cancer; TB: tuberculosis.

**Table 2:** List of c(RGD) multimeric radioligands α_v_β_3_-integrin cell and tumor models tested.

#	Name	Cell & Tumor Model	Ref.	Figure
**1**	[^18^F]FBOA-Dpr-HEG-c(RGDfE)	M21 Human melanoma U87MG human glioblastoma	[22,33]	Figure 2
**2**	[^18^F]FBOA-Dpr-K(HEG-c(RGDfE))_2_			
**3**	[^18^F]FBOA-Dpr-K{K[HEG-(c(RGDfE)]_2_}_2_			
**4**	[^18^F]FB-E[c(RGDyK)]_2_	HBCECs human brain capillary endothelial cells, U87MG	[34,35]	Figure 3
**5**	[^18^F]FP-E[c(RGDyK)]_2_ ^18^F-FP-RGD_2_	U87MG		
**6**	[^18^F]AlF-NOTA-E[c(RGDyK)]_2_ [^18^F]AlF-NOTA-RGD_2_	U87MG	[35,46]	
**7**	[^18^F]FB-PEG_3_-E[c(RGDyK)]_2_ [^18^F]FB-PRGD_2_	U87MG	[36,37]	
**8**	[^18^F]FP-PEG_3_-E[c(RGDyK)]_2_ [^18^F]FP-PRGD_2_	HCT116 human colon cancer, U87MG	[27,39,72,73,74]	
**9**	[^68^Ga]Ga-NOTA-PEG_3_-E[c(RGDyK)]_2_	U87MG	[38,39]	
**10**	[^18^F]AlF-NOTA-PEG_3_-E[c(RGDyK)]_2_ [^18^F]Alfatide I	U87MG, A549 adenocarcinomic human alveolar basal epithelial cells, PC-3 prostate cancer, LLC Lewis Lung Carcinoma	[38,39,40,41,76,77]	
**11**	[^18^F]FP-PEG_4_-E[c(RGDfK)]_2_ [^18^F]FP-PRGD_2_	U87MG, MDA-MB-435	[45]	Figure 4
**12**	[^68^Ga]Ga-NOTA-PEG_4_-E[c(RGDfK)]_2_	U87MG	[78]	
**13**	[^18^F]AlF-NOTA-PEG_4_-E[c(RGDfK)]_2_	U87MG	[46]	
**14**	[^18^F]AlF-NOTA-E[PEG_4_-c(RGDfK)]_2_, [^18^F]Alfatide II	U87MG, MDA-MB-435 human breast cancer	[43,44,46,52,53,54]	
**15**	[^68^Ga]Ga-NOTA-E[PEG_4_-c(RGDfK)]_2_ [^68^Ga]Ga-NOTA-PRGD_2_,	-	[51,79]	
**16**	[^18^F]FP-PEG_4_-E[PEG_4_-c(RGDfK)]_2_ [^18^F]FP-PPRGD_2_	U87MG, MDA-MB-435	[45]	
**17**	[^18^F]FP-PEG_2_-*β*-E[c(RGDyK)]_2_	A549, PC-3	[47]	Figure 5
**18**	[^18^F]FP-SAA-E[c(RGDyK)_2_	U87MG	[48]	Figure 6
**19**	[^18^F]FB-SAA-E[c(RGDyK)_2_			
**20**	NOTA-E[c(RGDyK)]_2_	-	[49]	Figure 7
**21**	[^68^Ga]Ga-NOTA-E[c(RGDyK)]_2_			
**22**	[^68^Ga]Ga-NOTA-Y-c(RGDyK)] (Y =2-(4-anilinyl-methyl)-4-(3-oxopropyl)thiazol-5(4H)-one)			
**23**	[^64^Cu]Cu-DOTA-E[c(RGDfK)]_2_	U87MG, MDA-MB-435	[55,56]	Figure 8
**24**	[^68^Ga]Ga-DOTA-E[c(RGDfK)]_2_	B16-F10-luc melanoma tumors, SK-RC-52, FaDu	[61,70]	
**25**	[^64^Cu]Cu-DOTA-E[c(RGDyK)]_2_	MDA-MB-435	[55]	
**26**	[^64^Cu]Cu-DOTA-E{E[c(RGDfK)]_2_}_2_	U87MG	[56,58]	
**27**	[^64^Cu]Cu-DOTA-E{E[c(RGDyK)]_2_}_2_	U87MG, c-neu onco-mice	[30]	
**28**	[^64^Cu]Cu-DOTA-E(E{E[c(RGDfK)]_2_}_2_)_2_	U87MG, c-neu onco-mice	[30]	
**29**	[^64^Cu]Cu-NOTA-PEG_4_-E[(PEG_2_-Tz-c(RGDfK)]_2_	HUVEC human umbilical vein endothelial cells	[57]	Figure 9
**30**	FITC-PEG_4_-E[PEG_2_-Tz-c(RGDfK)]_2_			
**31**	[^64^Cu]Cu-DOTA-PEG_4_-E[PEG_4_-c(RGDfK)]_2_	U87MG	[58]	
**32**	[^64^Cu]Cu-DOTA-G_3_-E[G_3_-c(RGDfK)]_2_			
**33**	[^64^Cu]Cu-AmBaSar-E[c(RGDyK)]_2_	U87MG	[59,60]	Figure 10
**34**	[^64^Cu]Cu-AmBaBaSar-c(RGDyK)_2_			
**35**	[^68^Ga]Ga-NOTA-E[PEG_4_-c(RGDfK)]_2_	U87MG: MDA-MB-435	[62]	Figure 11
**36**	[^68^Ga]Ga-NOTA-E[G_3_-c(RGDfK)]_2_	U87MG, MDA-MB-435, HT1080 fibrosarcoma	[62,64]	
**37**	[^68^Ga]Ga-NOTA-E[G_3_-c(CNGRC)]_2_	HT1080 fibrosarcoma	[64]	
**38**	[^68^Ga]Ga-NOTA-E[c(RGDyK)]_2_	U87MG	[66]	
**39**	[^68^Ga]Ga-NOTA-E{E[c(RGDyK)]_2_}_2_			
**40**	[^68^Ga]Ga-NODAGA-E[c(RGDyK)]_2_	U87MG, H727 human neuroendocrine	[67]	
**41**	[^64^Cu]Cu-NODAGA-E[c(RGDyK)]_2_			
**42**	[^68^Ga]Ga-FSC-[E-c(RGDfK)]_3_	M21 human melanoma, SK-RC-52 (human renal cell carcinoma), FaDu (human squamous cell carcinoma)	[68,70]	Figure 12
**43**	[^68^Ga]Ga-FSC-(CH_2_)-Tz-c(RGDfK)	U87MG, M21 human melanoma	[23]	
**44**	[^68^Ga]Ga-FSC-[(CH_2_)-Tz-c(RGDfK)]_2_			
**45**	[^68^Ga]Ga-FSC-[(CH_2_)-Tz-c(RGDfK)]_3_			
**46**	[^68^Ga]Ga-TRAP-PEG_4_-c(RGDfK)_3_	M21 human melanoma, SK-RC-52, FaDu	[69,70]	Figure 13
**47**	[^68^Ga]Ga-THP-c(RGDfK)_3_	SK-RC-52, FaDu	[70]	
